# Maternal Knowledge, Attitudes, and Practices Regarding Complementary Feeding Among Children Under 2 Years of Age: An Urban-Rural Comparison in Jharkhand, India

**DOI:** 10.7759/cureus.109844

**Published:** 2026-05-28

**Authors:** Kumari Asha Kiran, Neha Priya, Shalini Sunderam, Anuj K Anit, Dewesh Kumar, Anit Kujur

**Affiliations:** 1 Preventive Medicine, Rajendra Institute of Medical Sciences, Ranchi, IND; 2 Community Medicine, Rajendra Institute of Medical Sciences, Ranchi, IND

**Keywords:** behaviour change communication, bloom's classification, child nutrition, complementary feeding, jharkhand, kap pathway, maternal kap, urban–rural comparison

## Abstract

Background

Appropriate complementary feeding during the period of 6-24 months is essential for meeting the growing nutritional requirements of infants that cannot be met by breast milk alone after six months of age. Maternal knowledge, attitudes, and practices (KAP) are critical determinants of infant feeding behavior. Urban-rural disparities in education, socioeconomic status, and healthcare access may contribute to differences in these practices. This study aimed to assess and compare maternal KAP regarding complementary feeding in urban and rural areas of Ranchi district, Jharkhand, using a validated scoring framework.

Methods

A community-based cross-sectional study was conducted among 305 mothers of children aged 6-24 months in urban Doranda (n=107) and rural Ormanjhi (n=198), the field practice areas of the Department of Community Medicine, RIMS, Ranchi. Data were collected using a pre-tested semi-structured questionnaire. KAP was assessed using a Bloom’s cut-off-based scoring system: knowledge scores were classified as good (≥80%), moderate (60-79%), or poor (<60%); attitude as positive, neutral, or negative; and practice as good, moderate, or poor. Associations were tested using the chi-square test, and ORs were calculated to describe KAP pathways. Statistical significance was set at p<0.05.

Results

Overall, 63.3% of mothers had good knowledge, 58.0% held positive attitudes, and 54.1% demonstrated good complementary feeding practices. Urban mothers performed significantly better across all three domains (p=0.032, p=0.041, and p=0.006, respectively). Good knowledge was strongly associated with positive attitude (OR=5.35; 95% CI: 3.23-8.86; p<0.001) and good practice (OR=4.47; 95% CI: 2.60-7.68; p<0.001). Positive attitude showed the strongest association with good complementary feeding practices (OR=5.27; 95% CI: 3.05-9.11; p<0.001). Maternal education and household income were the most significant sociodemographic determinants of KAP (p<0.001 for both).

Conclusion

While general awareness was relatively high, significant gaps existed in practices, particularly in rural areas. The study demonstrates a sequential pathway from knowledge to attitude to practice, with socioeconomic factors as the primary underlying determinants. Targeted nutrition education and behavior change communication through frontline health workers are essential to translate knowledge into improved complementary feeding practices.

## Introduction

Complementary feeding, which usually begins at about six months of age, is the introduction of solid or semi-solid foods in addition to breast milk when breast milk alone is no longer adequate to meet all nutritional requirements [1]. The period between six months and two years of life is one of the most nutritionally vulnerable windows in childhood. Inadequate complementary feeding, including late or early food introduction, infrequent feeding, a lack of dietary diversity, and improper food preparation, remains one of the leading proximal causes of childhood undernutrition globally.

According to the WHO and UNICEF, optimal complementary feeding requires timely introduction at six months, adequate frequency, appropriate consistency, and sufficient dietary diversity, alongside the continuation of breastfeeding up to two years or beyond [2]. Despite these recommendations, suboptimal practices persist across low- and middle-income countries (LMICs). In India, the National Family Health Survey (NFHS-5) results indicated that 32.1%, 35.5%, and 19.3% of children under five years of age are underweight, stunted, and wasted, respectively [3]. Jharkhand is especially impacted, with 39.0% stunted, 24.3% wasted, and 39.5% underweight, above the national average [3]. These findings are corroborated by state reports that demonstrate nutritional challenges in Jharkhand [4].

Mothers’ knowledge, attitudes, and practices (KAP) toward infant feeding are important modifiable determinants of infant feeding practices and child development [5]. The KAP model suggests that knowledge leads to attitudes, which ultimately influence behaviors and practices. However, having knowledge does not guarantee practice, and a number of moderating factors, such as maternal education level, household wealth, cultural values, and urban-rural differences in access to health care, can interfere with the translation of information into practice. It is important to understand these relationships and the sociodemographic factors that influence them in order to develop targeted interventions.

Ranchi district in Jharkhand is a diverse community, including urban, rural, and tribal populations with diverse socioeconomic backgrounds and food cultures. While various schemes such as POSHAN Abhiyaan, Integrated Child Development Services (ICDS), and the Anganwadi system are in place, complementary feeding practices at the grassroots level remain inadequate. There is a dearth of evidence in this region that has comprehensively assessed maternal KAP using a scoring system and explored the pathway from knowledge to attitude to practice. Hence, this study was undertaken to evaluate maternal KAP about complementary feeding in the urban and rural settings of Ranchi district, using Bloom’s cut-off-based scoring method, to determine the sociodemographic correlates of KAP, and to examine associations between knowledge, attitude, and practice domains.

## Materials and methods

Study design and setting

The overall study duration for this thesis was from December 2023 to January 2026, which included synopsis development, ethical approval, data entry, analysis, thesis writing, and manuscript preparation. The study was conducted in two field practice areas of the Department of Community Medicine, Rajendra Institute of Medical Sciences (RIMS), Ranchi, Jharkhand, India. The Urban Health Training Centre, Doranda, was selected as the urban study area, while the Rural Health Training Centre, Ormanjhi, was selected as the rural study area to facilitate an urban-rural comparison. Data collection was carried out over a one-year period from June 2024 to June 2025 through household visits using a pre-tested semi-structured questionnaire. Each participant was assessed only once during the study period.

Study population and sampling

The study population included mothers of children aged 6-24 months who lived in the selected study areas. A multistage sampling strategy was used. In the first stage, one urban field practice area, Doranda, and one rural field practice area, Ormanjhi, of the Department of Community Medicine, RIMS, Ranchi, were purposively selected. In the second stage, households with eligible mother-child pairs were identified with the help of field health workers and Anganwadi registers. Eligible mothers were then recruited consecutively through household visits until the required sample size was reached: urban=107 and rural=198. Mothers who were ill, children who were very sick at the time of the survey, and mothers who were not at home after three visits to their households were not included.

Sample size

The sample size was calculated using the standard formula, N=Z²P(1−P)/d², assuming a 95% confidence level (Z=1.96), a prevalence (P) of appropriate complementary feeding practices of 51.3% based on a previous community-based Indian study by Varghese A et al. [6], and 6% absolute precision. The calculated sample size was approximately 267. After considering non-response and feasibility, a total of 305 mother-child pairs were enrolled in the study.

Data collection tool

Face-to-face interviews were conducted using a pre-tested semi-structured questionnaire based on standard Infant and Young Child Feeding (IYCF) guidelines and the literature. Experts from the Departments of Community Medicine and Pediatrics assessed the content validity. To determine clarity and feasibility, a pilot study was conducted among 30 mothers outside the study area, and changes were made where needed before the actual data collection.

The questionnaire covered socio-demographic characteristics and three domains: (i) knowledge, awareness of complementary feeding, correct age of initiation, recommended feeding frequency for different age groups, appropriate breastfeeding duration, and dietary diversity; (ii) attitude, perceived importance of complementary feeding, beliefs about breastfeeding sufficiency, food type preference, and recognition of dietary diversity; and (iii) practice, actual feeding behaviors, including timing of complementary food initiation, food type and consistency, feeding frequency, food preparation practices, hygiene, and continuation of breastfeeding.

KAP scoring and classification

Each KAP domain was scored using a structured, weighted scoring system based on Bloom’s cut-off criteria. Bloom’s cut-off points have been commonly applied in KAP studies for the categorization of knowledge, attitude, and practice scores [7]. For knowledge, responses to seven key questions were scored on a scale of 0-14 and classified as good (score 11-14; ≥80%), moderate (score 9-10; 60-79%), or poor (score 0-8; <60%) (Appendix 1). Attitude was assessed across four items on a scale of 0-5 points and rated as positive (score 4-5; ≥80%), neutral (score 3; 60-79%), or negative (score 0-2; <60%) (Appendix 2). Practice was scored across ten key feeding behavior variables on a scale of 0-10 and categorized as good (score 8-10; ≥80%), moderate (score 6-7; 60-79%), or poor (score 0-5; <60%) (Appendix 3). A correct response was scored as 1, or 2 for items with variable degrees of importance, and a wrong or “don’t know” answer received a score of 0.

Statistical analysis

Microsoft Excel was used for data entry, and IBM SPSS Statistics for Windows, Version 25.0 (IBM Corp., Armonk, NY, USA), was used for analysis. Frequencies and percentages were used for descriptive statistics. Differences in KAP between rural and urban areas and associations between sociodemographic factors and KAP categories were examined using the chi-square test. When expected cell counts were ≤5, Fisher’s exact test was used instead of the chi-square test. ORs and 95% CIs were used to express the pathways from knowledge to attitude, knowledge to practice, and attitude to practice. Statistically significant associations were defined as a p-value of <0.05.

Ethical clearance

Ethical approval was obtained from the Institutional Ethics Committee of RIMS, Ranchi (Reference: IEC-312/RIMS/CM/2023). Before data collection, each participant provided written informed consent. Anonymity and confidentiality were maintained throughout the study.

## Results

Socio-demographic profile

A total of 305 mothers were enrolled, 107 from the urban area, Doranda, and 198 from the rural area, Ormanjhi. The sociodemographic features of the study population are described in Table 1. The majority of mothers belonged to the 20-30 years age group (76.4%). Regarding educational status, secondary education was the most common level (34.7%), while only 4.6% were illiterate. Most mothers were housewives (78.0%). Half of the families had a monthly income between ₹5,000 and ₹10,000 (50.8%). Tribal mothers constituted a higher proportion in the rural area (29.3%) compared to the urban area (7.5%).

**Table 1:** Socio-demographic characteristics of study mothers (N=305). Socio-demographic characteristics of study mothers from rural (Ormanjhi) and urban (Doranda) areas, including age, religion, ethnicity, educational status, occupation, and monthly family income. Data are presented as frequencies (n) and percentages (%), calculated column-wise.

Variable	Category	Ormanjhi (Rural), n (%) (N=198)	Doranda (Urban), n (%) (N=107)	Total, n (%) (N=305)
Age of Mother	<20 years	10 (5.1)	3 (2.8)	13 (4.3)
	20-30 years	156 (78.8)	77 (72.0)	233 (76.4)
	31-40 years	31 (15.7)	25 (23.4)	56 (18.4)
	>40 years	1 (0.5)	2 (1.9)	3 (1.0)
Religion	Hindu	116 (58.6)	70 (65.4)	186 (61.0)
	Muslim	22 (11.1)	27 (25.2)	49 (16.1)
	Christian	5 (2.5)	6 (5.6)	11 (3.6)
	Others	55 (27.8)	4 (3.7)	59 (19.3)
Ethnicity	Tribal	58 (29.3)	8 (7.5)	66 (21.6)
	Non-tribal	140 (70.7)	99 (92.5)	239 (78.4)
Educational Status	Illiterate	8 (4.0)	6 (5.6)	14 (4.6)
	Primary	15 (7.6)	10 (9.3)	25 (8.2)
	Secondary	74 (37.4)	32 (29.9)	106 (34.8)
	Intermediate	56 (28.3)	31 (29.0)	87 (28.5)
	Graduate and above	45 (22.7)	28 (26.2)	73 (23.9)
Occupation	Housewife	150 (75.8)	88 (82.2)	238 (78.0)
	Manual labour	29 (14.6)	10 (9.3)	39 (12.8)
	Salaried employee (private/government)	15 (7.6)	8 (7.5)	23 (7.5)
	Business/self-employed	4 (2.0)	1 (0.9)	5 (1.6)
Monthly Family Income	< ₹5,000	24 (12.1)	12 (11.2)	36 (11.8)
	₹5,000-₹10,000	97 (49.0)	58 (54.2)	156 (50.8)
	₹10,000-₹20,000	69 (34.8)	29 (27.1)	98 (32.1)
	> ₹20,000	7 (3.5)	8 (7.5)	15 (4.9)
Total		198 (100)	107 (100)	305 (100)

Maternal knowledge regarding complementary feeding

Knowledge findings are presented in Table 2. Overall, 89.2% of mothers had heard about complementary feeding. Health workers, combined with family members, were the most common source of knowledge (57.7%). Correct knowledge of the recommended feeding frequency for infants aged 6-8 months, 2-3 times/day, was reported by 74.1% of mothers, with a higher proportion in the urban area (81.3%) than in the rural area (70.2%). For the 9-24-month age group, 63.3% correctly identified 3-4 feeds per day as the appropriate frequency. Knowledge of the recommended duration of breastfeeding, up to 2 years and beyond, was reported by 71.5% of mothers. Dietary diversity awareness was comparatively low, with only 37.4% recognizing its role in meeting nutritional requirements and supporting growth.

**Table 2:** Maternal knowledge regarding complementary feeding (N=305). Maternal knowledge regarding complementary feeding, including awareness, sources of information, recommended feeding frequency, duration of breastfeeding, and understanding of dietary diversity among rural and urban mothers. Data are presented as frequencies (n) and percentages (%). Multiple responses were allowed where indicated, and percentages were calculated column-wise.

Variable	Response	Ormanjhi, n (%)	Doranda, n (%)	Total, n (%)
Heard about complementary feeding	Yes	179 (90.4)	93 (86.9)	272 (89.2)
	No	19 (9.6)	14 (13.1)	33 (10.8)
Source of knowledge	Family + friends	30 (15.2)	26 (24.3)	56 (18.4)
	Health worker + family	132 (66.7)	44 (41.1)	176 (57.7)
	Health worker + family + friends	18 (9.1)	13 (12.1)	31 (10.2)
	Health worker + family + media	18 (9.1)	24 (22.4)	42 (13.8)
Feeding frequency, 6-8 months	1-2 times	40 (20.2)	13 (12.1)	53 (17.4)
	2-3 times	139 (70.2)	87 (81.3)	226 (74.1)
	3-4 times	18 (9.1)	3 (2.8)	21 (6.9)
	Don’t know	1 (0.5)	4 (3.7)	5 (1.6)
Feeding frequency, 9-24 months	1-2 times	8 (4.0)	1 (0.9)	9 (3.0)
	2-3 times	63 (31.8)	39 (36.4)	102 (33.4)
	3-4 times	127 (64.1)	66 (61.7)	193 (63.3)
	Don’t know	0 (0.0)	1 (0.9)	1 (0.3)
Duration of breastfeeding along with complementary feeding	Up to 2 years and beyond	141 (71.2)	77 (72.0)	218 (71.5)
	Until 1 year	52 (26.3)	27 (25.2)	79 (25.9)
	6-9 months	5 (2.5)	3 (2.8)	8 (2.6)
Importance of dietary diversity	Nutritional requirement + growth	66 (33.3)	48 (44.9)	114 (37.4)
	Growth only	57 (28.8)	31 (29.0)	88 (28.9)
	Nutrition + growth + deficiency prevention	51 (25.8)	19 (17.8)	70 (23.0)
	Growth + deficiency prevention	14 (7.1)	5 (4.7)	19 (6.2)
	Don’t know	10 (5.1)	4 (3.7)	14 (4.6)
Total		198 (100.0)	107 (100.0)	305 (100.0)

Maternal attitude toward complementary feeding

Attitude findings are presented in Table 3. The majority of mothers considered complementary feeding to be very important (52.8%) or moderately important (42.0%). A strongly positive attitude was seen regarding exclusive breastfeeding, with 86.9% believing that breast milk alone is sufficient for the first six months. An overwhelming 95.1% of mothers preferred homemade food over commercially packaged baby food. However, only 43.3% recognized the combined importance of feeding frequency and dietary diversity. A child’s refusal of food was the most commonly cited challenge to complementary feeding (47.9%).

**Table 3:** Maternal attitudes toward complementary feeding (N=305). Maternal attitudes toward complementary feeding, including perceived importance, beliefs regarding breastfeeding adequacy, preference for homemade versus packaged food, awareness of feeding practices, and challenges faced during feeding. Data are presented as frequencies (n) and percentages (%), calculated column-wise.

Variable	Response	Ormanjhi, n (%)	Doranda, n (%)	Total, n (%)
Importance of complementary feeding	Very important	98 (49.5)	63 (58.9)	161 (52.8)
	Moderately important	87 (43.9)	41 (38.3)	128 (42.0)
	Not important	13 (6.6)	3 (2.8)	16 (5.2)
Breast milk alone is sufficient for the first 6 months	Yes	167 (84.3)	98 (91.6)	265 (86.9)
	No	31 (15.7)	9 (8.4)	40 (13.1)
Homemade food is better than packaged baby food	Yes	187 (94.4)	103 (96.3)	290 (95.1)
	No	11 (5.6)	4 (3.7)	15 (4.9)
Importance of feeding frequency and dietary diversity	Yes	80 (40.4)	52 (48.6)	132 (43.3)
	Don’t know	118 (59.6)	55 (51.4)	173 (56.7)
Challenges faced during complementary feeding	Child refuses food	90 (45.5)	56 (52.3)	146 (47.9)
	Food is expensive	1 (0.5)	0 (0.0)	1 (0.3)
	Lack of knowledge about what to feed	15 (7.6)	6 (5.6)	21 (6.9)
	Lack of time	28 (14.1)	5 (4.7)	33 (10.8)
	No challenges faced	64 (32.3)	40 (37.4)	104 (34.1)
Total		198 (100.0)	107 (100.0)	305 (100.0)

Complementary feeding practices

Practice findings are summarized in Table 4. Early breastfeeding initiation within 30 minutes of birth was reported by 63.0% of mothers. Exclusive breastfeeding up to six months was practiced by 65.2%. Complementary feeding was initiated at the recommended age of six months by 64.3% of mothers, while 30.8% started before six months and 4.9% started after six months. Rice porridge was the most commonly introduced first food (61.0%). The majority of children (63.6%) received three or more feeds per day. Semi-solid food consistency was used by 59.0%. Continued breastfeeding alongside complementary feeding was practiced by 68.9%, with urban mothers showing a higher rate (76.6%) compared to rural mothers (64.6%). Pre-lacteal feeds were given by 9.2% of mothers. Handwashing combined with the use of clean utensils during food preparation was reported by 50.2%.

**Table 4:** Complementary feeding practices among study mothers (N=305). Complementary feeding practices among study mothers, including pre-lacteal feeding, initiation and duration of breastfeeding, timing of complementary feeding, types of foods introduced, feeding frequency, food consistency, hygiene practices, and continued breastfeeding. Data are presented as frequencies (n) and percentages (%). Multiple responses were allowed where applicable, and percentages were calculated column-wise.

Variable	Response	Rural, n (%)	Urban, n (%)	Total, n (%)
Pre-lacteal feed given	Yes	15 (7.6)	12 (11.2)	27 (8.9)
	No	183 (92.4)	95 (88.8)	278 (91.1)
Type of pre-lacteal feed	Animal milk	8 (4.0)	4 (3.7)	12 (3.9)
	Ghee	4 (2.0)	5 (4.7)	9 (3.0)
	Honey	3 (1.5)	3 (2.8)	6 (2.0)
Initiation of breastfeeding	Birth to 30 min	129 (65.2)	63 (58.9)	192 (63.0)
	30 min-4 hours	33 (16.7)	26 (24.3)	59 (19.3)
	4 hours-1 day after birth	20 (10.1)	12 (11.2)	32 (10.5)
	2-3 days after birth	16 (8.1)	6 (5.6)	22 (7.2)
Exclusive breastfeeding duration	Up to 6 months	132 (66.7)	67 (62.6)	199 (65.2)
	4-5 months	52 (26.3)	34 (31.8)	86 (28.2)
	2-3 months	14 (7.1)	6 (5.6)	20 (6.6)
Age at starting complementary feeding	At 6 months	131 (66.2)	65 (60.7)	196 (64.3)
	Before 6 months	61 (30.8)	33 (30.8)	94 (30.8)
	After 6 months	6 (3.0)	9 (8.4)	15 (4.9)
First complementary food introduced	Rice porridge	129 (65.2)	57 (53.3)	186 (61.0)
	Suji halwa	23 (11.6)	24 (22.4)	47 (15.4)
	Packaged baby food	29 (14.6)	17 (15.9)	46 (15.1)
	Dal/lentil soup	17 (8.6)	9 (8.4)	26 (8.5)
Complementary food prepared separately	Yes	86 (43.4)	52 (48.6)	138 (45.2)
	Sometimes	45 (22.7)	26 (24.3)	71 (23.3)
	No	67 (33.8)	29 (27.1)	96 (31.5)
Feeding separately or together with family	Separately	32 (16.2)	22 (20.6)	54 (17.7)
	Together with family	63 (31.8)	26 (24.3)	89 (29.2)
	Both	103 (52.0)	59 (55.1)	162 (53.1)
Feeding frequency per day	3-4 times or more	124 (62.6)	70 (65.4)	194 (63.6)
	2-3 times	66 (33.3)	35 (32.7)	101 (33.1)
	1-2 times	8 (4.0)	2 (1.9)	10 (3.3)
Dietary diversity in the last 24 hours	1-2 food groups	43 (21.7)	14 (13.1)	57 (18.7)
	3 food groups	126 (63.6)	67 (62.6)	193 (63.3)
	≥4 food groups	28 (14.1)	26 (24.3)	54 (17.7)
	Not reported/missing	1 (0.5)	0 (0.0)	1 (0.3)
Food consistency given	Semi-solid	117 (59.1)	63 (58.9)	180 (59.0)
	Semi-solid + solid	21 (10.6)	17 (15.9)	38 (12.5)
	Solid	35 (17.7)	9 (8.4)	44 (14.4)
	Watery/liquid	25 (12.6)	18 (16.8)	43 (14.1)
Hygiene during food preparation	Wash hands + clean utensils	90 (45.5)	63 (58.9)	153 (50.2)
	Wash hands + clean utensils + boiled water	69 (34.8)	29 (27.1)	98 (32.1)
	Wash hands only	22 (11.1)	8 (7.5)	30 (9.8)
	Clean utensils only	6 (3.0)	4 (3.7)	10 (3.3)
	Clean utensils + boiled water	11 (5.6)	3 (2.8)	14 (4.6)
Continued breastfeeding with complementary feeding	Yes	128 (64.6)	82 (76.6)	210 (68.9)
	No	70 (35.4)	25 (23.4)	95 (31.1)
Grand total		198 (100.0)	107 (100.0)	305 (100.0)

KAP classification: urban-rural comparison

KAP scores were classified using Bloom’s cut-off criteria, and the results are presented in Table 5. Overall, good knowledge was observed in 63.3% of mothers (urban: 70.1%; rural: 59.6%; p=0.032). A positive attitude was seen in 58.0% overall (urban: 64.5%; rural: 54.5%; p=0.041). Good complementary feeding practice was demonstrated by 54.1% of mothers overall (urban: 62.6%; rural: 49.5%; p=0.006). Urban mothers consistently outperformed rural mothers across all three KAP domains, with the most statistically significant urban-rural difference seen in the practice domain.

**Table 5:** Classification of KAP scores and urban-rural comparison (N=305). Distribution of maternal knowledge, attitude, and practice categories, with comparison between rural and urban populations based on predefined scoring criteria. Data are presented as frequencies (n) and percentages (%). Statistical comparisons were performed using the chi-square test, and a p-value of <0.05 was considered statistically significant. KAP: Knowledge, attitudes, and practices.

Component	Category	Ormanjhi, n (%)	Doranda, n (%)	Total, n (%)	χ²	p-value
Knowledge	Good	118 (59.6)	75 (70.1)	193 (63.3)	6.8	0.032
	Moderate	50 (25.3)	23 (21.5)	73 (23.9)		
	Poor	30 (15.1)	9 (8.4)	39 (12.8)		
Attitude	Positive	108 (54.5)	69 (64.5)	177 (58.0)	6.4	0.041
	Neutral	62 (31.3)	28 (26.2)	90 (29.5)		
	Negative	28 (14.1)	10 (9.3)	38 (12.5)		
Practice	Good	98 (49.5)	67 (62.6)	165 (54.1)	7.5	0.006
	Moderate	66 (33.3)	29 (27.1)	95 (31.1)		
	Poor	34 (17.2)	11 (10.3)	45 (14.8)		

Association of sociodemographic factors with KAP

The association of sociodemographic variables with KAP classification is shown in Table 6. Maternal education was strongly and significantly associated with all three KAP domains (p<0.001 for knowledge and practice; p=0.002 for attitude). Good knowledge increased progressively from 35.7% among illiterate mothers to 68.5% among those with graduate-level education. Good practice showed a similar gradient, rising from 28.6% among illiterate mothers to 63.0% among those with higher education. Monthly household income was equally influential, with good knowledge rising from 41.7% in the lowest income group to 80.0% in the highest group (p<0.001) and good practice rising from 27.8% to 75.6% (p<0.001). Occupation was also significantly associated with knowledge (p=0.038) and practice (p=0.021), with employed mothers demonstrating better outcomes. In contrast, maternal age, religion, and ethnicity were not significantly associated with any KAP domain (p>0.05), indicating that socioeconomic factors, rather than demographic or cultural identity markers, are the primary drivers of complementary feeding behavior.

**Table 6:** Association of sociodemographic characteristics with KAP (N=305). Association between sociodemographic variables and maternal knowledge, attitude, and practice levels. Data are presented as frequencies (n) and percentages (%). Statistical analysis was performed using the chi-square test, and a p-value of <0.05 was considered statistically significant. KAP: Knowledge, attitudes, and practices.

Variable	Category	Good knowledge, n (%)	χ² (K)	p-value (K)	Positive attitude, n (%)	χ² (A)	p-value (A)	Good practice, n (%)	χ² (P)	p-value (P)
Residence	Rural	118 (59.6)	4.6	0.032	108 (54.5)	4.18	0.041	98 (49.5)	7.5	0.006
	Urban	75 (70.1)			69 (64.5)			67 (62.6)		
Age of mother	<20 years	6 (46.2)	1.74	0.421	5 (38.5)	1.89	0.389	4 (30.8)	2.36	0.312
	20-30 years	150 (64.4)			135 (57.9)			120 (51.5)		
	31-40 years	34 (60.7)			32 (57.1)			30 (53.6)		
	>40 years	3 (100)			2 (66.7)			2 (66.7)		
Religion	Hindu	125 (67.2)	5.9	0.052	115 (61.8)	4.2	0.121	105 (56.5)	3.8	0.15
	Muslim	28 (57.1)			25 (51.0)			22 (44.9)		
	Christian	6 (54.5)			6 (54.5)			5 (45.5)		
	Others	34 (57.6)			31 (52.5)			33 (55.9)		
Ethnicity	Tribal	38 (57.6)	1.56	0.21	35 (53.0)	1.08	0.298	32 (48.5)	1.76	0.184
	Non-tribal	155 (64.9)			142 (59.4)			133 (55.6)		
Educational status	Illiterate	5 (35.7)	21.4	<0.001	5 (35.7)	14.8	0.002	4 (28.6)	24.6	<0.001
	Primary	10 (40.0)			12 (48.0)			10 (40.0)		
	Secondary	70 (66.0)			65 (61.3)			60 (56.6)		
	Intermediate	58 (66.7)			50 (57.5)			45 (51.7)		
	Graduate and above	50 (68.5)			45 (61.6)			46 (63.0)		
Occupation	Housewife	140 (58.8)	4.3	0.038	135 (56.7)	3.2	0.071	120 (50.4)	5.3	0.021
	Working, all others	53 (79.1)			42 (62.7)			45 (67.2)		
Monthly family income	<₹5,000	15 (41.7)	26.8	<0.001	12 (33.3)	15.6	0.001	10 (27.8)	32.4	<0.001
	₹5,000-₹10,000	95 (60.9)			90 (57.7)			80 (51.3)		
	₹10,000-₹20,000	65 (66.3)			60 (61.2)			58 (59.2)		
	>₹20,000	12 (80.0)			10 (66.7)			11 (73.3)		

Association between KAP domains

The associations between knowledge, attitude, and practice domains are shown in Table 7. Mothers with good knowledge had significantly higher odds of having a positive attitude (OR = 5.35; 95% CI: 3.23-8.86; p < 0.001). Good knowledge was also strongly associated with good practice, with mothers in the good knowledge category having 4.47 times the odds of demonstrating good feeding practices (OR = 4.47; 95% CI: 2.60-7.68; p < 0.001). The strongest association was observed between attitude and practice: mothers with a positive attitude had 5.27 times the odds of demonstrating good feeding practices compared to those with neutral or negative attitudes (OR = 5.27; 95% CI: 3.05-9.11; p < 0.001). These findings are consistent with the theoretical KAP model and demonstrate significant relationships between the knowledge, attitude, and practice domains.

**Table 7:** Association between KAP domains: ORs for observed associations (N=305). Association between knowledge, attitude, and practice domains assessed using ORs. Data are presented as frequencies (n) and percentages (%) where applicable. A p-value of <0.05 was considered statistically significant. KAP: Knowledge, attitudes, and practices.

Pathway	Exposure category	Outcome	n/N (%)	OR	95% CI	p-value
Knowledge → attitude	Good knowledge	Positive attitude	140/193 (72.5)	5.35	3.23-8.86	<0.001
	Moderate/poor knowledge	Positive attitude	37/112 (33.0)	Reference	-	-
Knowledge → practice	Good knowledge	Good practice	130/193 (67.4)	4.47	2.60-7.68	<0.001
	Moderate/poor knowledge	Good practice	35/112 (31.3)	Reference	-	-
Attitude → practice	Positive attitude	Good practice	125/177 (70.6)	5.27	3.05-9.11	<0.001
	Neutral/negative attitude	Good practice	40/128 (31.3)	Reference	-	-

## Discussion

In this community-based cross-sectional study, a validated Bloom’s cut-off scoring system and association analysis were used to assess the KAP of mothers regarding complementary feeding in urban and rural areas of Ranchi district, Jharkhand, among children aged 6 months to 2 years at the time of the study. The findings indicate that overall awareness of complementary feeding was relatively good, but significant gaps remained in attitudes and feeding practices, especially in rural areas. Significant associations were observed between the knowledge, attitude, and practice domains, while socioeconomic factors also showed important associations.

The outcomes of the present study showed that 63.3% of mothers had good knowledge about complementary feeding. This is similar to the study conducted in Kosovo by Berisha M et al., who found that a significant percentage of mothers knew the recommendations for complementary feeding, but there was a gap between knowledge and practice [8]. Similar findings have also been reported from several low- and middle-income countries, including Tanzania, Kenya, and the United Arab Emirates, where mothers demonstrated relatively good knowledge but suboptimal complementary feeding practices [9-11]. In Karnataka, Krishnegowda M et al. found that mothers’ knowledge regarding complementary feeding was good for almost three-fourths of the mothers, but appropriate feeding practices were significantly lower [12]. The prevalence of good knowledge was significantly lower among rural mothers than among urban mothers (59.6% vs. 70.1%, p=0.032). This difference may be attributed to lower educational status, limited access to health information, and fewer opportunities for nutrition counselling services in rural communities [12].

Overall, 58.0% of mothers had a positive attitude, and attitude was again significantly better among urban mothers than rural mothers (64.5% vs. 54.5%, p=0.041). The findings were similar to those of Gizaw AT et al. in Ethiopia, who found that almost half of rural mothers had positive attitudes toward complementary feeding practices [13]. Findings from other studies in Nepal indicated that maternal education and urban residence had significant effects on maternal attitudes and feeding behaviors [14]. There was a very positive attitude toward homemade food (95.1%) and exclusive breastfeeding (86.9%), indicating that public health messages under national nutrition programs have reached the community to a reasonable extent. However, awareness of feeding frequency and diversity among mothers was low, as only 43.3% of mothers recognized the importance of appropriate feeding frequency and dietary diversity, despite high awareness of complementary feeding initiation. This suggests that nutrition education programmes may focus on feeding initiation, while insufficient attention is given to nutrition quality, meal patterns, and food variety.

Overall, 54.1% of mothers had good complementary feeding practices. This proportion was relatively better compared with some rural and tribal areas in India. However, it still indicates that nearly half of the mothers practiced suboptimal feeding behaviors. Similar gaps between maternal knowledge and actual feeding practices were also reported in Rwanda [15]. The proportion of good practices in rural and urban areas was statistically different, with good practices seen in 49.5% of rural mothers and 62.6% of urban mothers (p=0.006). An important finding of the present study was that 30.8% of mothers started complementary foods before 6 months of age. Despite ongoing counselling, early complementary feeding is prevalent in many parts of India and has been linked to higher rates of gastrointestinal infections and allergy development, as well as interruption of exclusive breastfeeding [16]. This practice is known to be influenced by cultural beliefs, perceived inadequate breast milk supply, and advice from older family members.

The observed associations between KAP were an important finding of the present study. Mothers with good knowledge had significantly higher odds of positive attitudes toward complementary feeding (OR=5.35; 95% CI: 3.23-8.86; p<0.001) and good complementary feeding practices (OR=4.47; 95% CI: 2.60-7.68; p<0.001). The strongest association was observed between positive attitude and good feeding practices (OR=5.27; 95% CI: 3.05-9.11; p<0.001). These findings demonstrate statistically significant associations between the knowledge, attitude, and practice domains and are comparable with findings from previous studies [17]. This indicates that nutrition interventions need to shift from information provision to the application of nutrition-related behavior change communication strategies that target maternal perceptions and culture, feeding challenges, and household-level barriers.

The current study also showed that maternal education and family income were the sociodemographic factors most strongly associated with KAP regarding complementary feeding. There was a progressive increase in good knowledge from 35.7% among illiterate mothers to 68.5% among graduate mothers (p<0.001), and in good feeding practices from 28.6% to 63.0%, respectively. Such education gradients are consistently observed in other LMIC studies, including Indian studies [14]. Disparities in feeding practices were also significant on the basis of economic status, with good feeding practices rising from 27.8% among the poorest households to 75.6% among the wealthiest families (p<0.001). The results corroborate existing evidence showing that financial limitations have a considerable impact on food variety, meal adequacy, and food quality. Interestingly, maternal age, religion, and ethnicity were not found to have statistically significant associations with the KAP scores, which suggests that socioeconomic disadvantage may be more closely associated with feeding practices than cultural identity in this population.

The urban-rural differences observed across all three KAP domains suggest persistent inequities in infant and young child feeding practices in Jharkhand. Mothers in rural areas are likely to experience several disadvantages, such as low literacy, low family income, weak access to health services, and poor access to nutrition counselling services. Furthermore, some tribal and geographically marginalized populations may follow feeding habits that are not recommended. Mutuku JN et al. also found similar findings among pastoral communities in Kenya, with economic marginalization and geographic isolation being linked to poor complementary feeding KAP scores [10].

Strengths of the study

The results highlight the necessity of equity-based nutrition interventions for rural, tribal, and socioeconomically disadvantaged families. This study is one of the few community-based studies from Jharkhand that objectively assessed maternal KAP regarding complementary feeding using a structured Bloom’s cut-off-based KAP scoring system. The inclusion of urban and rural residents increased the public health relevance of the results and allowed comparisons between different sociodemographic settings. The results can be utilized to support nutrition education and behavior change communication initiatives in maternal and child health programmes at the district level, such as POSHAN Abhiyaan.

Limitations of the study

However, certain limitations should be considered while interpreting the findings. Due to the cross-sectional study design, causal relationships between KAP domains could not be established. The study relied on self-reported responses, which may be subject to recall and social desirability bias. Some subgroup analyses involved small cell frequencies, despite the use of Fisher’s exact test where appropriate. Additionally, since the study was conducted in two selected field practice areas of Ranchi district, the findings may not be fully generalizable to the entire population of Jharkhand or India.

## Conclusions

This study showed that while general awareness of complementary feeding was high among mothers in Ranchi, significant and measurable gaps existed in both attitudes and practices, particularly in rural areas. Using a Bloom’s cut-off-based KAP scoring system, we found that only 54.1% of mothers demonstrated good complementary feeding practices overall, with a statistically significant urban-rural disparity. Significant associations were observed between the knowledge, attitude, and practice domains, with positive attitude showing the strongest association with good complementary feeding practices (OR=5.27). Maternal education and household income were the sociodemographic factors most strongly associated with KAP, while ethnicity, religion, and age were not significantly associated with KAP. These findings highlight the need for targeted behavior change communication interventions that address not only knowledge gaps but also attitudinal barriers, delivered through frontline workers such as ASHAs and Anganwadi workers using culturally adapted tools, with priority given to low-income, low-literacy, and rural households.

## Appendices

Appendix 1 

**Table 8:** Scoring criteria for assessment of maternal knowledge regarding complementary feeding. Variables and scoring criteria used for the assessment of maternal knowledge regarding complementary feeding among mothers of children aged 6-24 months. Correct or appropriate responses were assigned scores according to the predefined scoring criteria, while incorrect or inappropriate responses were assigned a score of 0. Total scores were used to categorize the level of knowledge based on predefined cutoff values. ANM: Auxiliary Nurse Midwife; ASHA: Accredited Social Health Activist.

Variable	Response Category	Score
Heard about complementary feeding	Yes	1
	No	0
Source of knowledge	Health workers (ANM/ASHA)	1
	Family members	1
	Friends/neighbours	1
	Traditional beliefs	1
	Media (TV/internet/social media)	1
Age of introducing complementary feeding	At 6 months	1
	Before or after 6 months / Don’t know	0
Frequency for 6-8-month-old child	1 time	0
	2-3 times	1
Frequency for 9-24-month-old child	3-4 times	2
	2-3 times	1
	1-2 times	0
Duration of breastfeeding	6-9 months	0
	Till 1 year	1
	Up to 2 years and beyond	2
Importance of food variety	Nutritional requirements	1
	Healthy growth and development	1
	Prevention of deficiency diseases	1
	Don’t know	0

Appendix 2 

**Table 9:** Scoring criteria for assessment of maternal attitudes toward complementary feeding. Attitude variables and scoring criteria used for the assessment of maternal attitudes toward complementary feeding among mothers of children aged 6-24 months. Responses were assigned scores based on their appropriateness, with higher scores indicating more favorable attitudes. Total attitude scores were calculated and categorized into predefined levels for analysis.

Variable	Response Category	Score
Importance of complementary feeding	Very important	2
	Moderately important	1
	Not important	0
Breast milk alone sufficient for the first 6 months	Yes	1
	No	0
Homemade food is better than packaged food	Yes	1
	No	0
Feeding frequency and dietary diversity are important	Yes	1
	Don’t know	0

Appendix 3 

**Table 10:** Scoring criteria for assessment of maternal practices regarding complementary feeding. Practice variables and scoring criteria used for the assessment of complementary feeding practices among mothers of children aged 6-24 months. Appropriate practices were assigned a score of 1, while inappropriate practices were assigned a score of 0. The total practice score was calculated and used to categorize mothers into predefined levels of practice for analysis.

Variable	Response Category	Score
Pre-lacteal feed given	No	1
	Yes	0
Initiation of breastfeeding	Birth to 30 minutes	1
	After 30 minutes	0
Exclusive breastfeeding duration	Up to 6 months	1
	Less than 6 months	0
Age of starting complementary feeding	At 6 months	1
	Before or after 6 months	0
First complementary food introduced	Rice porridge/dal/lentil soup	1
	Packaged baby food	0
Complementary food prepared separately	Yes	1
	Sometimes/No	0
Feeding frequency per day	3-4 times or more	1
	Less than 3 times	0
Food consistency given	Semi-solid/semi-solid + solid	1
	Watery/liquid/only solid	0
Hygiene during food preparation	Washing hands + clean utensils (± boiled water)	1
	Other/no handwashing	0
Continued breastfeeding with complementary feeding	Yes	1
	No	0
